# Genome-wide unraveling of the *AUX/IAA* family in *Avena sativa* L. with observations on seedling root growth and stress resilience

**DOI:** 10.3389/fpls.2026.1836152

**Published:** 2026-06-09

**Authors:** Qian Li, Yuanhang Wang, Fang Liu, Siqi Yuan, Yutao Yuan, Ma Bai, Zihan Zhao, Qingping Zhou, Chen Chen, Youjun Chen

**Affiliations:** 1 Sichuan Zoige Alpine Wetland Ecosystem National Observation and Research Station, Southwest Minzu University, Chengdu, China; 2 Tibetan Plateau Ethnic Medicinal Resources Protection and Utilization Key Laboratory of National Ethnic Affairs Commission of the People’s Republic of China, Southwest Minzu University, Chengdu, China; 3 Sichuan Provincial Qiang-Yi Medicinal Resources Protection and Utilization Technology and Engineering Laboratory, Southwest Minzu University, Chengdu, China; 4 Xiexiong Agricultural and Animal Husbandry Comprehensive Service Center of Dingqing, Changdu, China

**Keywords:** abiotic stress, Aux/IAA gene family, auxin, *Avena sativa*, gene expression, molecular breeding, root development

## Abstract

The *Aux/IAA* gene family plays a central role in auxin signaling and plant development. To investigate the underlying molecular mechanisms, we performed a genome-wide identification of the *Aux/IAA* gene family in *Avena sativa*. In this study, we observed that exogenous IAA exerts a dual regulatory effect on *A. sativa* root growth—promoting elongation at low concentrations (5–50 µM) and inhibiting it at high concentrations (100–200 µM)— highlighting the dose-dependent nature of auxin action. To investigate the underlying molecular mechanisms, we performed a genome-wide identification of the *Aux/IAA* gene family in *Avena sativa*. A total of 40 *AsIAA* genes were identified and phylogenetically classified into five subfamilies. Structural and motif analyses confirmed evolutionary conservation, while duplication analysis revealed that segmental and tandem events contributed to family expansion. Expression profiling showed tissue-specific patterns, with *AsIAA13/15/19* preferentially expressed in flowers and *AsIAA17* enriched in leaves. Under IAA, PEG, NaCl, and temperature treatments, six selected *AsIAA* genes (*AsIAA6/11/13/15/17/19*) exhibited distinct and stress-specific expression changes, suggesting their potential involvement in both developmental regulation and environmental adaptation. These findings suggest their potential involvement in both developmental regulation and environmental adaptation. This study provides a comprehensive characterization of the *AsIAA* gene family and offers candidate genes for functional studies and molecular breeding in *A. sativa*.

## 1 Introduction

Auxin (indole-3-acetic acid, IAA) orchestrates plant development and stress adaptation through a hallmark feature: concentration-dependent bidirectional regulation, wherein low concentrations promote growth while high concentrations exert inhibitory effects (Wang et al., 2025a). This regulatory duality is decoded by the canonical *Aux/IAA-ARF* signaling module. Under low auxin conditions, *Aux/IAA* repressors dimerize with auxin response factors (ARFs) and recruit the transcriptional co-repressor TOPLESS, thereby suppressing auxin-responsive genes (Kumar et al., 2026; Li et al., 2023). Upon auxin accumulation, IAA stabilizes the interaction between F-box receptors TIR1/AFB and Aux/IAA proteins, triggering ubiquitin-mediated degradation of the repressors via the 26S proteasome (Das et al., 2024; Reddy et al., 2024).

Degradation of Aux/IAA proteins releases ARFs from repression, enabling them to activate or repress transcription of target genes in a context-specific manner (Lavy et al., 2016). This de-repression mechanism effectively converts extracellular auxin concentration gradients into precise, dynamic transcriptional outputs—underpinning the hormone’s ability to orchestrate diverse developmental and physiological responses across space and time (Zeng et al., 2024). Moreover, auxin action is highly context-dependent—varying across plant species, tissue types, developmental stages, and environmental conditions—thereby exhibiting pronounced spatiotemporal specificity (Li et al., 2023; Wei et al., 2021).

In forage crops, fine-tuning auxin signaling holds dual promise for enhancing both biomass yield and forage quality. IAA modulates vegetative growth, secondary cell wall biosynthesis, and nitrogen assimilation, directly impacting crude protein content and neutral detergent fiber (NDF) levels—two key determinants of forage digestibility and nutritional value (Zeng et al., 2024; Zhang et al., 2023). Furthermore, IAA precisely governs critical developmental transitions, such as heading and flowering (Liu et al., 2017; Wojcikowska and Gaj, 2017). Recent studies have revealed that the Aux/IAA protein family not only regulates plant growth and development but also functions as a central signaling hub that integrates hormonal cues with environmental adaptation (Shani et al., 2017). A landmark study in Science (2025) demonstrated that drought-induced reactive oxygen species (ROS) trigger redox-dependent multimerization of IAA3, which enhances its interaction with TOPLESS and suppresses lateral root branching—a xerobranching response that conserves water under deficit conditions (Roy et al., 2025). In rice, *OsIAA20* positively regulates drought and salt tolerance through crosstalk with abscisic acid (ABA) signaling (Zhang et al., 2021), while in tomato, *SlIAA9* modulates drought tolerance by affecting ROS scavenging capacity (Cao et al., 2025). In wheat, overexpression of *TaIAA15-1A* enhances drought tolerance by regulating the ABA signaling pathway (Su et al., 2023). Aux/IAA proteins also integrate environmental signals through physical interactions with other signaling components, such as the Drought-induced 19 (Di19) protein, and function in concert with other phytohormones and transcription factors to balance growth and stress responses (Kumar et al., 2026; Maitra Majee et al., 2020). For instance, accelerated flowering in alfalfa reduces forage biomass, implying that Aux/IAA activity may indirectly influence forage yield (Wolabu et al., 2023). Conversely, the high expression of *DgIAA17* in orchard grass during the heading stage—and the marked acceleration of flowering upon its overexpression—demonstrates its direct role in controlling flowering time (Wang et al., 2025b). These findings collectively highlight the *Aux/IAA* family as a promising molecular target for improving forage stress tolerance without compromising productivity.

Despite these advances, the *Aux/IAA* gene family remains completely uncharacterized in *A. sativa*. A globally important cereal and forage crop valued for its high protein content, favorable fiber composition, and remarkable adaptability to marginal environments (Warchoł et al., 2023; Huang et al., 2025). *A. sativa* possesses a complex allohexaploid genome (AACCDD, 2n = 6x = 42) with an estimated size of ~11 Gb and a repeat content exceeding 86%, which has historically hindered systematic functional genomic studies (J. Hilli and Kapoor, 2023; Peng et al., 2022). This complex genome may have led to expansion and functional divergence of the *Aux/IAA* family compared with diploid relatives, but this has never been examined. Recent breakthroughs—including the release of a gap-free reference genome, a pan-genome of 33 cultivated and wild oat lines, and a genus-level super-pangenome comprising 35 *Avena* accessions—have finally rendered oat amenable to genome-wide investigations (Kamal et al., 2022). Capitalizing on these resources, several recent studies have identified candidate genes governing drought resistance, nutritional quality, and agronomic traits (Song et al., 2025; Ma et al., 2025). However, the *Aux/IAA* family has been conspicuously overlooked, leaving critical questions unanswered: How many *AsIAA* genes exist in the *A. sativa* allohexaploid genome? How are they distributed across the A, C, and D subgenomes? What are their tissue-specific expression patterns, and how do they respond to exogenous auxin and abiotic stresses such as drought and salinity? Addressing these questions is essential to understand how polyploidization has shaped auxin signaling networks in *A. sativa* and to identify functional alleles that could be harnessed for breeding.

Here, we performed a genome-wide identification and comprehensive characterization of the *AsIAA* gene family in *A. sativa*. This study employed a multi-omics integrated analysis strategy: By integrating phylogenetic analysis, gene structure examination, chromosomal localization, promoter cis-element prediction, and expression profiling under IAA treatment, polyethylene glycol (PEG)-induced drought, and NaCl stress, we delineated the evolutionary landscape and functional divergence of *AsIAA* genes. Our results not only fill a critical gap in auxin signaling research in *A. sativa* but also provide a suite of candidate genes for molecular breeding aimed at optimizing root growth, forage yield, quality, and stress resilience in this understudied yet agriculturally valuable crop.

## 2 Materials and methods

### 2.1 Plant materials and experimental treatments

The stress-tolerant *A. sativa* cultivar ‘Qingyan No. 2’ was used in this study. For root phenotype analysis, plump and uniform seeds were surface-sterilized (75% ethanol for 1 min, 5% sodium hypochlorite for 5 min, rinsed 3–5 times with sterile water) and then sown on MS medium supplemented with various concentrations of IAA (0, 5, 10, 50, 100, or 200 μM, pH 5.8–6.0), with 0 μM serving as the control (CK). Each treatment consisted of three independent biological replicates, with five plants per replicate. Seedlings were grown in a growth chamber under a photoperiod of 14 h light/8 h dark, a light intensity of 200 μMol·m^-2^·s^-1^, temperatures of 20 ± 2 °C, and a relative humidity of 60-70%. After 7 days, root elongation and plant architecture were recorded. For tissue-specific expression, roots, stems, leaves and flowers were collected separately at early flowering stage (three biological replicates). For hormone and stress treatments, plants at three-leaf stage (outdoor greenhouse) were treated with IAA (50 and 100 mg/L), polyethylene glycol (PEG) (10% and 20%), NaCl (150 and 300 mmol/L), and temperature (4 °C and 37 °C) (three biological replicates). Each treatment was applied to three pots (30 mL/pot); controls received distilled water. One hour post-treatment, whole plants were harvested, snap-frozen in liquid nitrogen, and stored at -80 °C for RNA extraction and qRT-PCR (Quantitative Real-Time PCR).

### 2.2 Identification of the *AsIAA* gene family

The genome sequence of hexaploid *A. sativa* (cultivar “Sang”) was retrieved from the Ensembl Plants database (https://plants.ensembl.org/index.html). The Hidden Markov Model (HMM) profile corresponding to the *IAA* domain was obtained from the Pfam database (https://pfam.xfam.org/). Using HMMER 3.1 software (http://hmmer.janelia.org) with an e-value threshold of < 10^-5^, potential *IAA* family genes were initially screened from the *A. sativa* genome database. Candidate genes were further validated via the Conserved Domain Database (CDD) of the National Center for Biotechnology Information (NCBI) (https://www.ncbi.nlm.nih.gov/cdd/) to retain only those containing the canonical *IAA* conserved domain. Additionally, *TaIAA* genes were used as query sequences to perform BLAST searches against the *A. sativa* genome database, followed by re-validation using the NCBI CDD. Finally, the results from the two screening approaches were merged, and redundant entries were eliminated to obtain the final set of *AsIAA* family genes.

### 2.3 Subcellular localization, protein properties, chromosomal localization, and phylogenetic tree construction of *AsIAA* family proteins

The potential subcellular localization of *AsIAA* proteins was predicted using the BUSCA tool (http://busca.biocomp.unibo.it), a dedicated subcellular localization prediction platform (Savojardo et al., 2018). The physicochemical properties of AsIAA proteins were analyzed via the protein parameter calculation module integrated in TBtools-II software (Chen et al., 2023), while the same software was employed to generate chromosomal localization maps of *AsIAA* genes. Based on prior research, protein sequences of *IAA* domain-containing genes from *Arabidopsis thaliana*, *Oryza sativa*, *Triticum aestivum*, and *Zea mays* were retrieved from the Ensembl Plants database. The complete IAA sequences of these species were filtered to remove redundant or incomplete sequences. Multiple sequence alignment was then performed using the MUSCLE algorithm in MEGA 11 software (Tamura et al., 2021). Subsequently, phylogenetic analysis of the aligned protein sequences was conducted using IQ-TREE 2 software (Minh et al., 2020), with the best-fit substitution model automatically selected by ModelFinder, and a maximum likelihood (ML) phylogenetic tree was constructed with a bootstrap value of 1,000.

### 2.4 Analysis of collinearity, gene structure, conserved motifs, and conserved domains

The analysis of whole-genome duplication (WGD)/segmental duplication and tandem duplication events in the genome of *A. sativa* was performed using the collinearity analysis tool MCScanX. This tool was employed to align *IAA* homologous genes between *A. sativa* and selected species (*Arabidopsis*, rice, and wheat). TBtools-II software was used to generate collinearity relationship maps and chromosomal localization plots of genes between *A. sativa* and the aforementioned species. The structural features (including introns, exons, and untranslated regions) of *A. sativa AsIAA* genes were parsed based on the gene annotation file in GFF3 format. The conserved motif composition of *AsIAA* proteins was analyzed using the MEME online platform (http://meme-suite.org/) with the following parameters: maximum number of motifs set to 10, and optimal motif width ranging from 26 to 50 amino acids.

### 2.5 Prediction of Cis-acting elements

The 2000-bp sequences upstream of the transcription start sites (TSSs) of *AsIAA* family genes were extracted using TBtools-II. Cis-acting elements within the promoter regions of *AsIAA* genes were predicted and analyzed via the PlantCARE online platform (http://bioinformatics.psb.ugent.be/webtools/plantcare/html/). Visualization of the prediction results was implemented using TBtools-II and GraphPad Prism 9.5 software.

### 2.6 qRT-PCR expression analysis of *AsIAA* genes

The wheat *TaIAA15-1A* gene, previously shown to be involved in the regulation of flowering and drought tolerance, was used as a query sequence in BLAST searches against the *A. sativa* genome. The top six homologous *AsIAA* genes (*AsIAA6/11/13/15/17/19*) were selected for preliminary expression analysis. Total RNA was extracted from tissue samples using a plant RNA extraction kit. Genomic DNA contamination was removed by DNase I treatment according to the manufacturer’s instructions. A no-reverse transcription control (no-RT) was included for each sample to verify the absence of genomic DNA amplification. Subsequently, cDNA was synthesized from 1 μg of DNase-treated RNA via reverse transcription. qRT-PCR was performed using SYBR Green Master Mix on a real-time PCR system, with three biological and three technical replicates. Gene-specific primers (**Supplementary Table 1**) were designed using Primer3 and synthesized commercially. *AsEIF4A* was used as the internal reference, and relative expression levels were calculated using the 2^-ΔΔCt^ method. Each 20 μL reaction contained 2 μL cDNA template, 10 μL SYBR Green Master Mix, and 0.2 μM primers. The thermal cycling program was: 95 °C for 30 s; 40 cycles of 95 °C for 10 s and 60 °C for 30 s; followed by melting curve analysis.

### 2.7 Statistical analysis

All experiments were performed with three independent biological replicates, each containing three technical replicates. The sample size (three biological replicates per group) was determined based on preliminary experiments and common practice in the field. Data are presented as mean ± standard deviation (SD). Normality was assessed using the Shapiro–Wilk test, and homogeneity of variances was evaluated using Levene’s test. For data that met both normality and homoscedasticity assumptions, comparisons between treatment groups and the control (CK) were conducted using one-way analysis of variance (ANOVA) followed by Dunnett’s *post hoc* test for multiple comparisons. For data that violated normality or homoscedasticity, the Kruskal–Wallis test with Bonferroni correction was applied. All comparisons were based on independent samples. Significance levels are indicated as *p < 0.05, **p < 0.01, and ***p < 0.001. Statistical analyses were performed using SPSS (version 26.0; IBM Corp., Armonk, NY, USA) and GraphPad Prism 9.0 (GraphPad Software, San Diego, CA, USA).

## 3 Results

### 3.1 Low IAA concentrations promote while high concentrations inhibit root elongation in *A. sativa*

**Figure 1:**
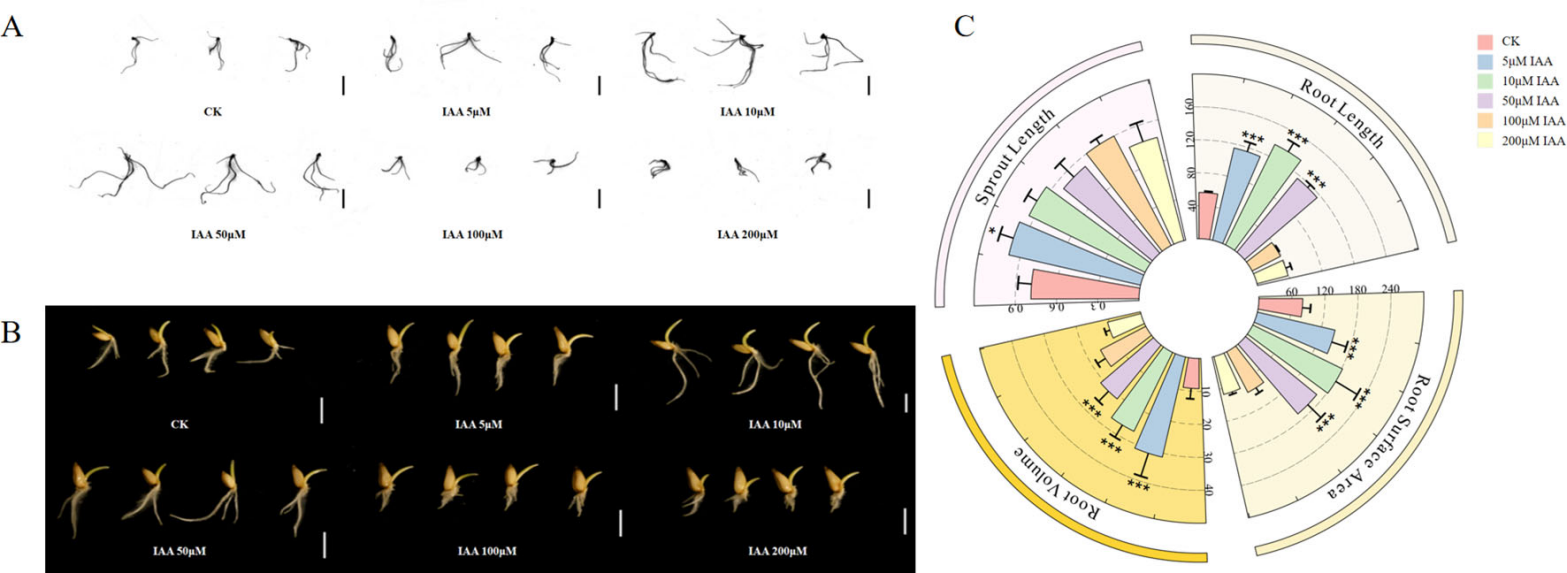
Dose−dependent biphasic effect of exogenous IAA on root growth in A. sativa seedlings. **(A)** Representative scans of A. sativa roots treated with varying IAA concentrations (0, 5, 10, 50, 100, 200 µM) for 7 days. Scale bar = 1* cm*. **(B)** Detailed morphology of root tips under each concentration. **(C)** Quantitative analysis of root length, sprout length, root surface area, and root volume. Data are shown as mean ± SD (n = 5). Asterisks indicate statistically significant differences compared to the control (CK) using one−way ANOVA with Dunnett’s test (*p < 0.05, **p < 0.01, ***p < 0.001).

To establish a phenotypic basis for subsequent gene expression analyses, we first examined the effects of exogenous IAA at varying concentrations on root growth of *A. sativa* seedlings. Consistent with the well-documented biphasic action of auxin, low IAA concentrations (5-50 μM) promoted root elongation, whereas higher concentrations (100-200 μM) inhibited it (**Figures 1A–C**). At lower concentrations (5 μM, 10 μM, and 50 μM IAA), *A. sativa* root elongation was significantly promoted, characterized by increased root length, more robust root architecture, and expanded root surface area and root volume. The promoting effect on root surface area peaked at 10 μM, where root expansion and growth were most pronounced. As IAA concentrations increased to 100–200 μM, root elongation was gradually inhibited, and root surface area decreased, indicating that supra-optimal IAA concentrations exerted a negative impact on root growth. Regarding shoot growth, all IAA treatments led to increased shoot length; however, no statistically significant differences were observed among the tested concentrations. Root volume reached its peak at 5 μM and then showed a decreasing trend with increasing IAA concentrations. These phenotypes confirm the functional conservation of auxin dose-response in *A. sativa* and provide a rationale for prioritizing *AsIAA* genes potentially involved in mediating this dose-dependent response.

### 3.2 Genome-wide identification reveals 40 *AsIAA* genes with divergent physicochemical properties and duplication-driven expansion

**Figure 2:**
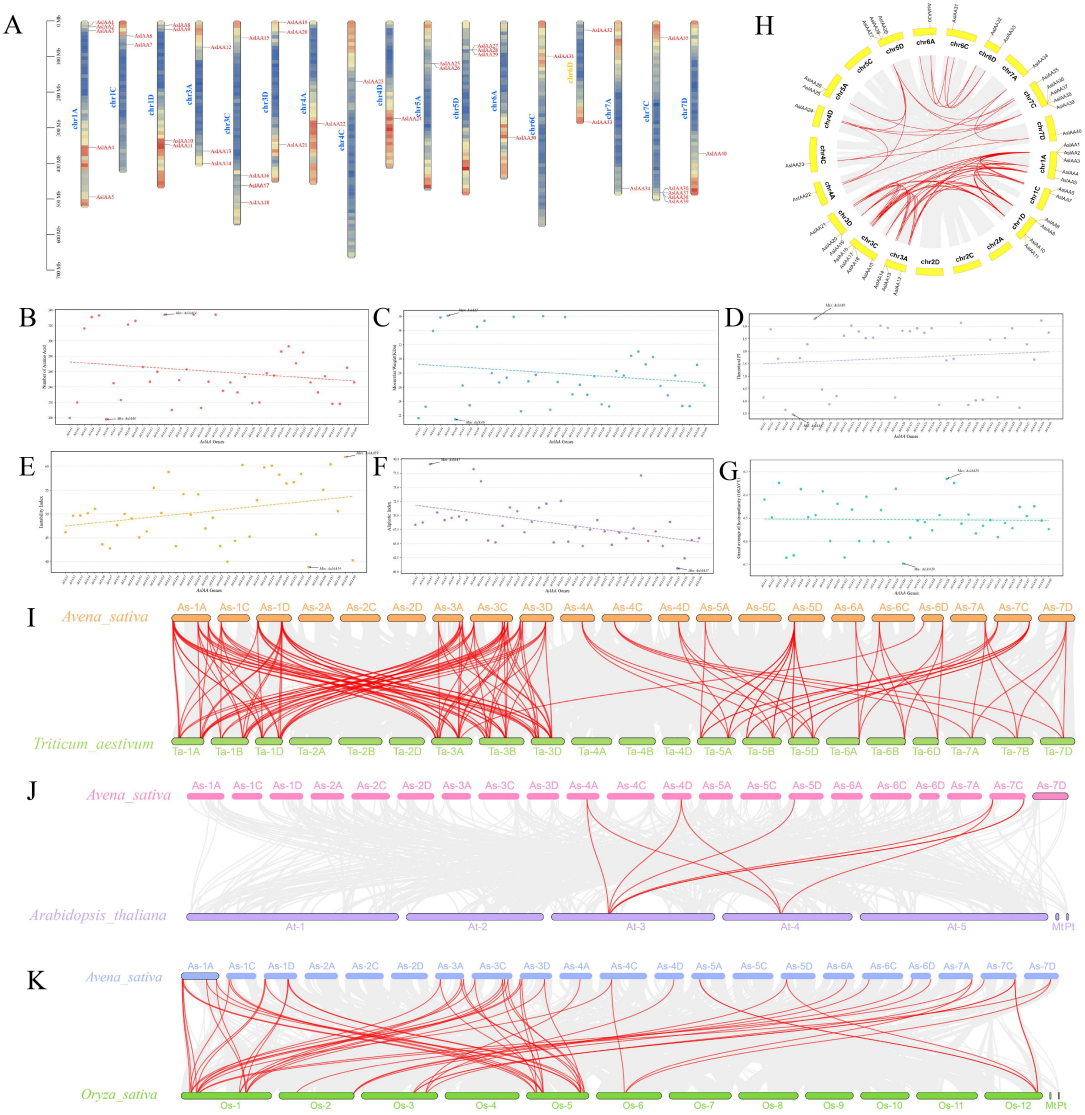
Genome−wide identification, physicochemical properties, and duplication events of the *AsIAA* gene family. **(A)** Chromosomal localization. **(B)** Number of Amino Acids. **(C)** Molecular weight (KDa). **(D)** Theoretical PI. **(E)** Instability Index. **(F)** Aliphatic Index. **(G)** Grand average of hydropathicity (GRAVY). **(H)** Collinearity analysis of *AsIAA* genes, red lines indicate syntenic *AsIAA* gene pairs. **(I)** Collinearity analysis of *AsIAA* genes with *T. aestivum*. **(J)** Collinearity analysis of *AsIAA* genes with *A. thaliana*. **(K)** Collinearity analysis of *AsIAA* genes with *O. sativa.*.

A total of 40 non-redundant *AUX/IAA* genes were identified in the A. sativa genome and designated *AsIAA1* to *AsIAA40* (**Supplementary Table 2**). Chromosomal mapping showed uneven distribution across 17 chromosomes, with chromosomes 1A and 7C harboring five each, while no *AsIAA* genes were detected on chromosomes 2A/2C/2D or 5C (**Figure 2A**). The protein lengths of the 40 *AsIAA* family members varied from 198 amino acids (*AsIAA6*) to 334 amino acids (*AsIAA14/18/21*) (**Figure 2B**). Their molecular weights ranged from 21.47 kDa (*AsIAA6*) to 36.09 kDa (*AsIAA5*) (**Figure 2C**). The theoretical isoelectric points (pI) spanned 5.47 (*AsIAA5*) to 9.3 (*AsIAA8*): 13 *AsIAA* family members with a theoretical pI < 7 were classified as acidic proteins, while 27 *AsIAA* family members with a theoretical pI > 7 were categorized as basic proteins (**Figure 2D**). The instability index ranged from 38.88 (*AsIAA34*) to 61.94 (*AsIAA39*); only *AsIAA23* and *AsIAA34* were predicted to be stable proteins, whereas the remaining members were unstable (**Figure 2E**). The aliphatic index varied from 60.6 (*AsIAA37*) to 79.08 (*AsIAA3*). The grand average of hydropathicity (GRAVY) values ranged from –0.696 (*AsIAA20*) to –0.331 (*AsIAA26*), indicating that all AsIAA proteins are hydrophilic. Subcellular localization prediction revealed that all AsIAA proteins are localized to the nucleus. The pI versus GRAVY scatter plot showed no correlation between charge and hydrophilicity, and the relationship between aliphatic index and instability index highlighted structural heterogeneity (**Figures 2F, G**). To understand the evolutionary relationships of *IAA* genes in *A. sativa*, gene duplication events within the *A. sativa* genome were analyzed. To ensure reliable identification of collinear regions, whole-genome synteny blocks among the three subgenomes of *A. sativa* were first defined using MCScanX implemented in TBtools. In the resulting synteny map, gray lines represent collinear blocks between genomes (i.e., the syntenic backbone), while red lines highlight collinear relationships specifically among *AsIAA* gene family members. A total of 73 gene duplication events (segmental and tandem) were identified among *AsIAA* genes, particularly on chromosomes 1A, 5A/5D, and 7C (**Figure 2H**). Interspecies synteny analysis revealed 99,025 collinear gene pairs between wheat and *A. sativa*, accounting for 38.10% of the *A. sativa* genome; 3,807 collinear gene pairs between *Arabidopsis* and *A. sativa* (2.12%); and 60,264 collinear gene pairs between rice and *A. sativa* (32.03%) (**Figures 2I–K**). The higher number of homologous genes between *A. sativa* and wheat may be attributed to both species belonging to the Triticeae tribe within the Poaceae family. These findings provide important insights into the expansion and evolutionary origin of the *AsIAA* gene family, supporting expansion driven by polyploidization.

### 3.3 Phylogenetic clustering, conserved motifs, and intron–exon structures define clade-specific features

**Figure 3:**
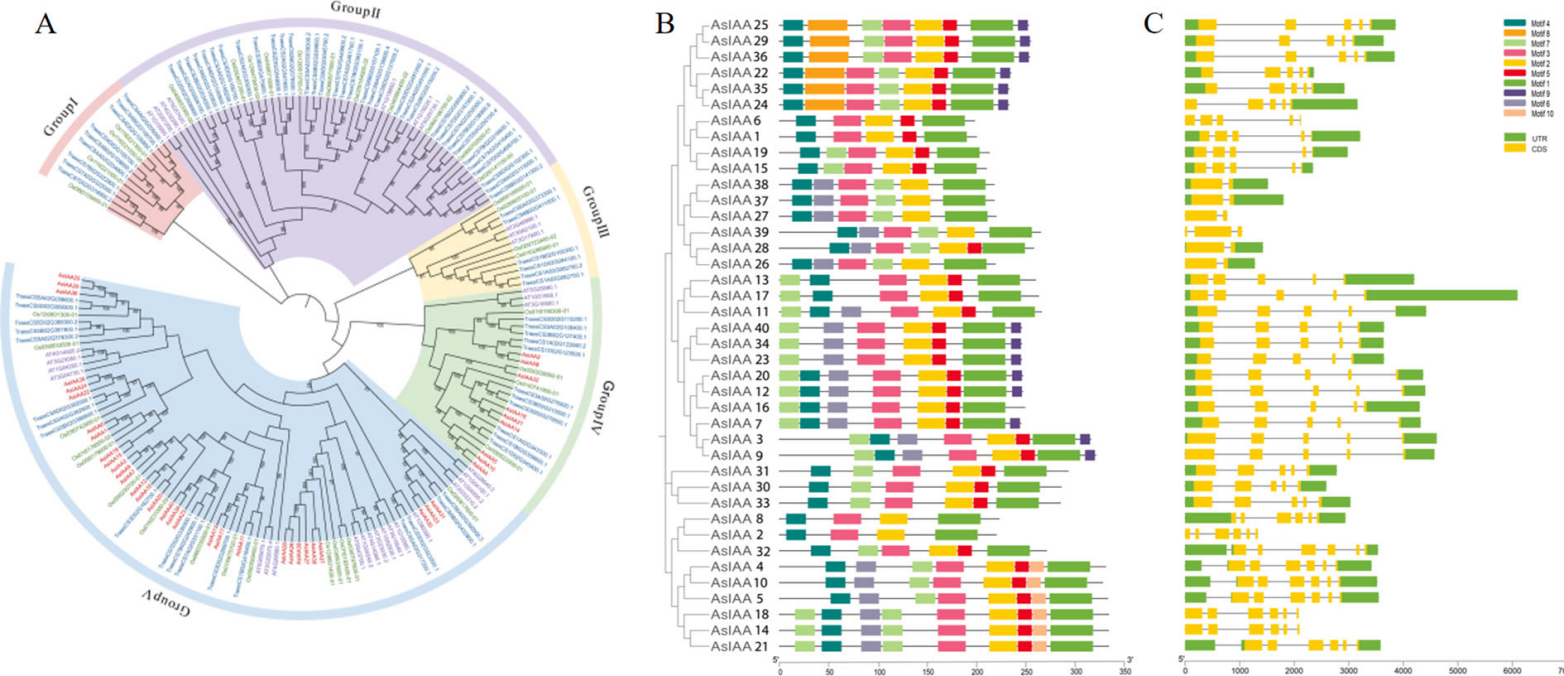
Phylogenetic relationships, conserved motifs, and gene structures of *AsIAA* genes. **(A)** Maximum likelihood phylogenetic tree of *AsIAA* genes with *A. thaliana (At), O. sativa (Os)* and *T. aestivum (Ta) IAA* family genes. **(B)** Phylogenetic tree and motif compositions of *AsIAA* genes. **(C)** Gene structure of *AsIAA* genes.

To gain insights into the evolutionary dynamics of *IAA* genes in *A. sativa*, we constructed a maximum likelihood (ML) phylogenetic tree using 189 full-length IAA protein sequences from four species: 32 from *A. thaliana*, 38 from *O. sativa*, 79 from *T. aestivum*, and 40 from *A. sativa* (**Figure 3A**). These 189 proteins were clustered into five distinct subfamilies: Subfamily I (13 members), Subfamily II (53 members), Subfamily III (13 members), Subfamily IV (27 members, including 9 AsIAAs), and Subfamily V (83 members, including 31 AsIAAs). Notably, no *AsIAA* genes were detected in Subfamilies I, II, or III—indicating that these gene lineages may have been lost during *A. sativa* evolution, while being retained in wheat, rice, and *Arabidopsis*. This observation reflects the species-specific patterns of gene evolution across these taxa. To investigate the evolutionary dynamics of the *AsIAA* gene family in *A. sativa*, we analyzed exon-intron structures and characterized conserved motif compositions (**Figures 3B, C**). Gene structure analysis revealed that intron numbers ranged from 2 to 6 across the family: 9 genes (*AsIAA2/4/5/8/10/14/18/21/32*) contained 6 introns, while 3 genes (*AsIAA26-28*) harbored only 2 introns. Conserved motif analysis identified 10 distinct motifs (Motif 1-10) with clade-specific distribution patterns. Notably, all 40 *AsIAA* genes retained Motifs 1 and 2, confirming these two motifs as the core functional elements of the *AsIAA* gene family. The clade-specific distribution of other motifs and variable intron numbers imply functional divergence among subclades.

### 3.4 Promoter regions of *AsIAA* genes are enriched in hormone- and stress-responsive Cis-elements

**Figure 4:**
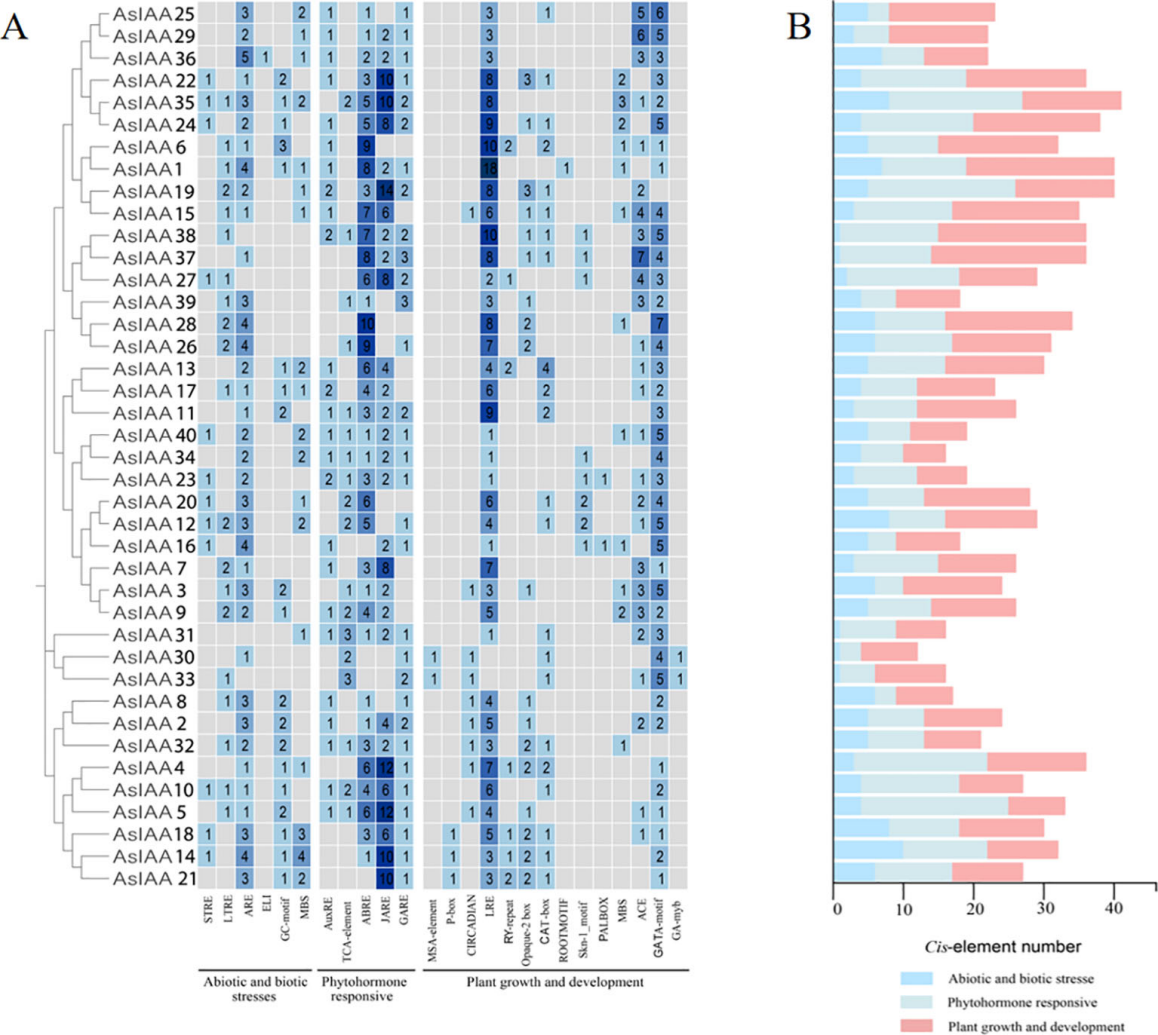
Enrichment of cis−acting elements in the promoter regions of *AsIAA* genes. **(A)** Number of *cis*-elements of *AsIAA* genes. **(B)** Bar chart of the number of cis-acting elements in *AsIAA* genes.

Through comprehensive analysis of the promoter regions of the *AsIAA* gene family (2000 bp upstream sequences), a total of 25 distinct cis-acting regulatory elements were predicted (**Figure 4**). These elements were functionally classified into three major categories: (i) abiotic/biotic stress-responsive elements (STRE, LTRE, ARE, ELI, GC-motif, MBS); (ii) plant hormone-responsive elements (AuxRE, TCA-element, ABRE, JARE, GARE); and (iii) growth- and development-related elements (MSA-element, P-box, CIRCADIAN, LRE, RY-repeat, Opaque-2 box, CAT-box, ROOTMOTIF, Skn-1_motif, PALBOX, ACE, GATA-motif, GA-myb). Notably, MBS appears in both stress- and development-related categories, reflecting its dual functional roles reported in prior literature. Quantitative motif profiling revealed that six elements (ARE, ABRE, JARE, GARE, LRE, and GATA-motif) were detected in ≥30 *AsIAA* genes, raising the possibility of broad conservation across the family. In contrast, nine elements (EIL, MSA-element, P-box, CIRCADIAN, RY-repeat, ROOTMOTIF, Skn-1_motif, PALBOX, and GA-myb) occurred in < 10 genes each, hinting at potential lineage- or subfamily-specific regulatory functions. EIL was exclusively identified in *AsIAA36*, while ROOTMOTIF was uniquely present in *AsIAA1*. According to the prediction, promoter element enrichment analysis further showed that *AsIAA14* contains the greatest number of abiotic/biotic stress-responsive elements; *AsIAA5* and *AsIAA19* harbor the highest abundance of hormone-responsive elements; and *AsIAA37* exhibits the most extensive complement of growth- and development-related elements. This combinatorial promoter architecture supports the integration of auxin signaling with diverse environmental cues.

### 3.5 Six *AsIAA* genes show tissue-preferential and stress-specific expression patterns

**Figure 5:**
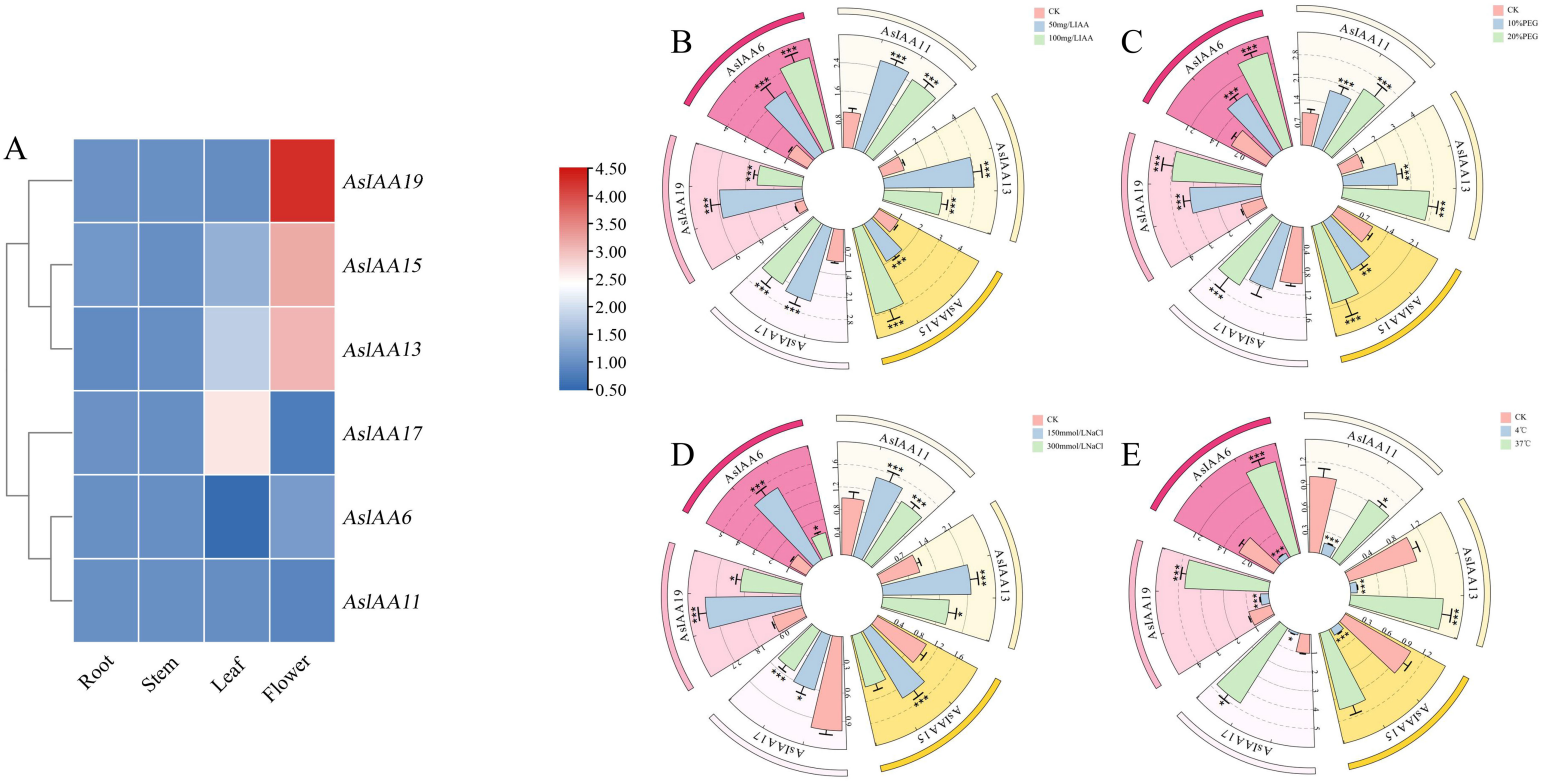
Tissue−preferential and multi−stress expression profiles of six candidate *AsIAA* genes. **(A)** Relative expression levels of *AsIAA6/11/13/15/17/19* in root, stem, leaf, and flower tissues. Red indicates high expression, and blue indicates low expression. **(B–E)** Relative expression levels of *AsIAA* genes in *A. sativa* seedlings under different treatment conditions. **(B)** IAA treatment, **(C)** PEG stress, **(D)** NaCl stress, **(E)** Temperature stress (4 °C and 37 °C). Data are presented as mean ± SD (n = 9). Asterisks indicate significant differences compared to the control (CK) or to root tissue (A) using one−way ANOVA with Dunnett’s test (*p < 0.05, **p < 0.01, ***p < 0.001).

qRT-PCR was employed to systematically characterize the tissue-specific expression patterns of six *AsIAA* genes (*AsIAA6/11/13/15/17/19*) across four *A. sativa* tissues: roots, stems, leaves, and floral organs (**Figure 5A**). All six genes were constitutively expressed in all examined tissues; however, their transcript abundance exhibited pronounced tissue specificity. Notably, *AsIAA13/15/19* displayed significantly elevated expression in floral organs, implicating potential roles in the regulation of *A. sativa* flower development. In contrast, *AsIAA13/17* showed preferential accumulation in leaves, suggesting its involvement in leaf morphogenesis or physiological homeostasis. By comparison, *AsIAA6* exhibited the lowest expression level among all tissues specifically in leaves. Collectively, these findings suggest that *AsIAA13/15/17/19* display robust and divergent tissue-specific expression signatures, implying possible functional specialization in organ-specific developmental programs that warrants further experimental validation.

To dissect the regulatory mechanisms underlying the response of the *AsIAA* gene family to endogenous auxin (IAA) and abiotic stressors—including NaCl (salt stress), PEG (drought-mimicking stress), and temperature extremes—the six candidate genes were subjected to qRT-PCR analysis (**Figures 5B–E**). Under IAA treatment, all six *AsIAA* genes exhibited significant transcriptional upregulation relative to the untreated control (CK). Notably, *AsIAA19* displayed the highest expression level at both 50 mg/L and 100 mg/L IAA. Under PEG-induced osmotic stress, transcript levels of all six genes were significantly elevated compared to CK, with *AsIAA19* again showing maximal induction at 20% PEG. Under NaCl stress, *AsIAA6/11/13/15/19* were significantly upregulated relative to CK; *AsIAA6* reached peak expression at 150 mmol/L NaCl. In contrast, *AsIAA17* was significantly downregulated under all tested NaCl concentrations. Under temperature stress, differential regulation was observed: all six genes showed significant transcriptional repression at 4 °C (cold stress), whereas *AsIAA6/13/17/19* exhibited significant upregulation at 37 °C (heat stress). Collectively, these stimulus-specific expression patterns demonstrate that *AsIAA6/11/13/15/17/19* function as context-dependent signaling integrators—capable of discriminating between and coordinately responding to endogenous auxin signaling and distinct abiotic stress cues—thereby supporting their specialized roles in adaptive growth regulation.

## 4 Discussion

### 4.1 Effects of IAA on root development phenotypes and significance of systematic identification of the *A. sativa AsIAA* gene family

As a central phytohormone, auxin (IAA) regulates plant growth through a well-characterized concentration-dependent dual effect: low concentrations promote cell elongation and organ growth, whereas high concentrations trigger inhibition and developmental reprogramming (Cho et al., 2024). This canonical response was recapitulated in *A. sativa* roots—exogenous IAA promoted root elongation at lower concentrations but inhibited it at higher concentrations. Notably, root surface area and root volume peaked at different IAA doses (10 μM and 5 μM, respectively), suggesting that auxin may regulate radial and longitudinal growth via partially distinct pathways. This phenotypic dichotomy validates the functional conservation of auxin signaling in A. sativa root development and provides a rationale for prioritizing *AsIAA* genes potentially involved in mediating this dose-dependent response. Recent genome-wide studies have expanded our understanding of *Aux/IAA* family evolution in Poaceae: 36 *HvIAA* genes in barley (Shi et al., 2020), 92 *SsIAA* in sugarcane (Huang et al., 2024), and 30 *DgIAA* in orchardgrass (Wang et al., 2023). In contrast, comprehensive characterization in *A. sativa*—a globally important allohexaploid cereal—has remained scarce. Leveraging the high-quality reference genome (cv. ‘Kanota’), we systematically identified 40 non-redundant *Aux/IAA* genes, named *AsIAA1–AsIAA40*. These encode proteins with variable lengths (198–334 aa) and molecular weights (14.5-47.8 kDa), consistent with subfunctionalization or neofunctionalization.

All predicted AsIAA proteins are hydrophilic, contain conserved nuclear localization signals (NLS), and are predicted to localize to the nucleus—consistent with their role as transcriptional corepressors in auxin signaling (Feng et al., 2024; Kubalova et al., 2025). Promoter cis-element analysis revealed that *AsIAA* promoters are enriched not only in canonical auxin-responsive elements (e.g., TGA-box, AuxRE) but also in stress- and hormone-responsive motifs, including ABRE (ABA), MYB/MYC binding sites (drought/cold), G-box (light/stress), and JA-responsive elements (e.g., CGTCA-motif). This combinatorial architecture suggests that *AsIAA* genes may integrate endogenous auxin gradients with environmental cues, contributing to transcriptional reprogramming during root development under abiotic stress. Future experimental validation of these predicted cis-elements is needed, using yeast one-hybrid and dual-luciferase reporter assays.

### 4.2 Expression of core candidate *AsIAA* genes suggests their potential roles in organ development and response to auxin and abiotic stresses

The auxin signaling pathway regulates plant growth and development (Cance et al., 2022; Caumon and Vernoux, 2023; Das et al., 2024), but its molecular basis for balancing organogenesis and environmental plasticity remains unresolved. To prioritize candidates, we identified six *AsIAA* genes orthologous to the wheat flowering regulator *TaIAA15-1A*. Their tissue-specific expression showed flower enrichment for *AsIAA13/15/19* and leaf preference for *AsIAA17*. This compartmentalized expression suggests possible functional specialization—namely, the formation of distinct transcriptional modules governing floral organ identity and leaf morphogenesis, respectively—but this remains to be tested. Such spatial signal decoding via differential *Aux/IAA* expression has been documented across diverse species, including *Medicago truncatula* (Zhang et al., 2024), *Salvia miltiorrhiza* (Huang et al., 2023), and *T. aestivum* (Jia et al., 2021), underscoring its evolutionary conservation within the Poaceae and beyond.

To assess their roles in signal integration, we examined transcriptional responses of six *AsIAA* genes to hormonal and abiotic stresses by qRT-PCR. Expression patterns varied with treatment and concentration, suggesting dose-dependent stress responsiveness. Notably, *AsIAA17* was consistently repressed by NaCl, while the other five showed more complex responses. PEG stress upregulated all six genes, with *AsIAA19* showing the highest induction. Cold (4 °C) generally repressed expression, whereas heat (37 °C) induced a subset of genes. These stimulus-specific patterns suggest that the examined *AsIAA* genes may integrate endogenous and environmental signals. However, expression data were collected only at 1 h post-treatment; future time-course experiments are needed to capture full dynamics. In addition, expression profiling should be extended to all 40 *AsIAA* members, and future work should characterize *AsIAA–ARF* interactions, degradation dynamics, and reporter activity *in vivo*. This paradigm is evolutionarily conserved: *PmIAA* genes in *Prunus mume* integrate auxin, salt, and drought signals (Cheng et al., 2022); specific *SsIAA* members in *Saccharum spontaneum* are co-induced by cold and salinity (Huang et al., 2024); and *TrIAA18* in *Trifolium repens* responds synergistically to both IAA and NaCl (Qi et al., 2024). Together, these findings support the *Aux/IAA* family as a conserved module coordinating development and stress responses.

### 4.3 Hypotheses for future gene-editing-based crop improvement targeting *AsIAA* genes

This study leveraged the canonical auxin dose–response phenotype in *A. sativa* roots as a phenotypic anchor to achieve a comprehensive, genome-wide identification of the *Aux/IAA* gene family in *A. sativa*, yielding 40 high-confidence members designated *AsIAA1–AsIAA40*. By integrating phylogenetic analysis, tissue-specific expression profiling, and multi-stress transcriptional responsiveness, we prioritized six core candidate genes (*AsIAA6/11/13*/*15/17/19*) for functional characterization. Their spatially restricted expression—particularly flower-enriched *AsIAA13*/*15*/*19*, and leaf-preferential *AsIAA17*—together with stimulus- and dose-dependent transcriptional dynamics under IAA, salt, drought, and temperature stresses, establishes a robust foundation for deciphering auxin-mediated coordination of developmental programming and environmental adaptation in *A. sativa*.

Grounded in the evolutionarily conserved *Aux/IAA–ARF* signaling module (Feng et al., 2024; Li et al., 2023) and empirically validated gene–trait associations, we tentatively propose three potential molecular design strategies for future validation. These are speculative suggestions based on sequence homology and expression patterns, rather than experimentally confirmed outcomes. Specifically: (1) editing Domain II of flower-enriched *AsIAA13/15/19* might delay flowering without compromising root growth, as suggested by Arabidopsis gain-of-function mutants (Kubalová et al., 2024); (2) targeted mutagenesis of degradation domains in *AsIAA14/18/21* could potentially optimize tillering for dense planting, based on rice *OsIAA6* orthology (Jung et al., 2015); and (3) precise editing of the *OsIAA7* ortholog *AsIAA32* may enhance grain yield (Qiu et al., 2025).

Future validation will entail (i) generation and phenotypic characterization of CRISPR-edited *AsIAA* lines; (ii) systematic annotation of the *A. sativa ARF* gene family to reconstruct complete *Aux/IAA–ARF* interaction networks; and (iii) multi-location field trials to assess agronomic performance—including flowering time, tiller number, root system architecture, stress resilience, and grain yield—under contrasting management intensities. Collectively, these efforts will translate fundamental insights into deployable tools for precision *A. sativa* breeding, establishing *AsIAA* genes as functionally validated, trait-linked targets for next-generation molecular design.

## 5 Conclusions

This study provides the first comprehensive characterization of the *AUX/IAA* gene family in *A. sativa*, integrating phenotypic, genomic, and expression analyses. The identification of 40 *AsIAA* genes, combined with the tissue- and stress-specific expression patterns of six selected *AsIAA* genes, provides candidate genes and preliminary expression information for future studies on auxin-mediated growth regulation and stress adaptation. Notably, the dose-dependent root responses to IAA and the expression of *AsIAA* genes under hormonal and abiotic stresses highlight their potential as targets for molecular breeding. Future functional validation of candidate genes, particularly *AsIAA13/15/17/19*, will be essential to determine whether they can serve as targets for developing *A. sativa* varieties with optimized root architecture and enhanced stress resilience.

## Data Availability

Publicly available datasets were analyzed in this study. This data can be found here: http://plants.ensembl.org/index.html.

## Supplementary material

Supplementary Table 1 Primer information for qPCR used in this study.

Supplementary Table 2 Physicochemical properties of *AsIAA* genes.

Supplementary Table 3 Orthologs of *AsIAA* family genes in different species.

Supplementary Table 4 Ct values and 2^-^ΔΔCT values of genes under different treatment conditions and in different tissues.
