# Supplementary material for: Genome-wide unraveling of the AUX/IAA family in Avena sativa L. with observations on seedling root growth and stress resilience
Source: Front Plant Sci. 2026 Jun 9;17:1836152. doi: 10.3389/fpls.2026.1836152 (PMC13286757; doi:10.3389/fpls.2026.1836152)
Supplement: Supplementary Table 1 — Primer information for qPCR used in this study. [file Table1.docx]

TableS2. Physicochemical properties of *AsIAA* genes.

| Gene Name | Gene ID | Chromosome Locations | Number of Amino Acid | Molecular Weight(KDa) | Theoretical PI | Instability Index | Aliphatic Index | Grand average of hydropathicity (GRAVY) | Subcelluar Localization |
| --- | --- | --- | --- | --- | --- | --- | --- | --- | --- |
| AslAA1 | AVESA.00010b.r2.1AG0075520.1 | Chr1A:12212366-12215574 | 200 | 21.66 | 6.15 | 46.21 | 68.35 | -0.42 | Nucleus. |
| AslAA2 | AVESA.00010b.r2.1AG0074920.1 | Chr1A:16515687-16517022 | 220 | 23.26 | 8.88 | 49.61 | 68.77 | -0.497 | Nucleus. |
| AslAA3 | AVESA.00010b.r2.1AG0073550.1 | Chr1A:27652822-27657434 | 316 | 33.96 | 7.7 | 49.68 | 79.08 | -0.349 | Nucleus. |
| AslAA4 | AVESA.00010b.r2.1AG0041140.1 | Chr1A:354704229-354707646 | 331 | 35.84 | 5.65 | 50.17 | 70.51 | -0.671 | Nucleus. |
| AslAA5 | AVESA.00010b.r2.1AG0009360.1 | Chr1A:494197726-494201276 | 333 | 36.09 | 5.47 | 51.08 | 69.19 | -0.66 | Nucleus. |
| AslAA6 | AVESA.00010b.r2.1CG0108450.1 | Chr1C:41931417-41933540 | 198 | 21.47 | 7.73 | 43.66 | 69.55 | -0.375 | Nucleus. |
| AslAA7 | AVESA.00010b.r2.1CG0105810.1 | Chr1C:66174928-66179246 | 245 | 26.23 | 8.28 | 42.82 | 69.8 | -0.496 | Nucleus. |
| AslAA8 | AVESA.00010b.r2.1DG0193750.1 | Chr1D:12561885-12564824 | 223 | 23.47 | 9.3 | 47.64 | 69.19 | -0.487 | Nucleus. |
| AslAA9 | AVESA.00010b.r2.1DG0192380.1 | Chr1D:23351094-23355665 | 321 | 34.52 | 6.47 | 49.99 | 78.19 | -0.384 | Nucleus. |
| AslAA10 | AVESA.00010b.r2.1DG0159960.2 | Chr1D:336218427-336221947 | 326 | 35.36 | 5.88 | 49.06 | 76.04 | -0.599 | Nucleus. |
| AslAA11 | AVESA.00010b.r2.1DG0155920.1 | Chr1D:346918384-346922802 | 266 | 27.97 | 6.2 | 45.12 | 65.53 | -0.439 | Nucleus. |
| AslAA12 | AVESA.00010b.r2.3AG0421800.1 | Chr3A:73669982-73674384 | 247 | 26.67 | 8.62 | 46.35 | 65.22 | -0.67 | Nucleus. |
| AslAA13 | AVESA.00010b.r2.3AG0442950.1 | Chr3A:365485162-365489360 | 260 | 27.34 | 9.02 | 55.49 | 68.15 | -0.463 | Nucleus. |
| AslAA14 | AVESA.00010b.r2.3AG0449320.1 | Chr3A:399835995-399838089 | 334 | 35.92 | 8.8 | 50.22 | 71.41 | -0.599 | Nucleus. |
| AslAA15 | AVESA.00010b.r2.3CG0463110.1 | Chr3C:47374035-47376379 | 210 | 22.61 | 8.53 | 58.8 | 70.76 | -0.408 | Nucleus. |
| AslAA16 | AVESA.00010b.r2.3CG0492950.2 | Chr3C:434096012-434100312 | 249 | 26.83 | 8.55 | 43.28 | 67.03 | -0.6 | Nucleus. |
| AslAA17 | AVESA.00010b.r2.3CG0496400.1 | Chr3C:462317549-462323642 | 263 | 27.76 | 9.02 | 54.19 | 68.82 | -0.465 | Nucleus. |
| AslAA18 | AVESA.00010b.r2.3CG0503170.1 | Chr3C:509748334-509750415 | 334 | 36.02 | 8.94 | 49.82 | 71.38 | -0.603 | Nucleus. |
| AslAA19 | AVESA.00010b.r2.3DG0517130.1 | Chr3D:3467765-3470745 | 213 | 22.83 | 6.15 | 54.14 | 72.07 | -0.374 | Nucleus. |
| AslAA20 | AVESA.00010b.r2.3DG0523010.1 | Chr3D:30942293-30946657 | 247 | 26.74 | 8.82 | 46.98 | 65.22 | -0.696 | Nucleus. |
| AslAA21 | AVESA.00010b.r2.3DG0550250.1 | Chr3D:347964946-347968531 | 334 | 35.88 | 8.81 | 49.24 | 72.57 | -0.585 | Nucleus. |
| AslAA22 | AVESA.00010b.r2.4AG0606550.1 | Chr4A:288627473-288629833 | 235 | 25.01 | 8.92 | 43.25 | 65.32 | -0.511 | Nucleus. |
| AslAA23 | AVESA.00010b.r2.4CG1288180.1 | Chr4C:169442536-169446181 | 246 | 26.34 | 8.74 | 39.98 | 67.93 | -0.518 | Nucleus. |
| AslAA24 | AVESA.00010b.r2.4DG0750210.1 | Chr4D:273847659-273850819 | 233 | 24.92 | 8.92 | 44.36 | 64.59 | -0.553 | Nucleus. |
| AslAA25 | AVESA.00010b.r2.5AG0814630.1 | Chr5A:119538715-119542573 | 253 | 27.56 | 5.85 | 60.3 | 67.51 | -0.487 | Nucleus. |
| AslAA26 | AVESA.00010b.r2.5AG0814670.1 | Chr5A:119763526-119764805 | 219 | 23.6 | 7.63 | 45.26 | 69.18 | -0.331 | Nucleus. |
| AslAA27 | AVESA.00010b.r2.5DG0993850.1 | Chr5D:81496424-81497196 | 220 | 23.28 | 7.7 | 52.9 | 67.14 | -0.349 | Nucleus. |
| AslAA28 | AVESA.00010b.r2.5DG0993840.1 | Chr5D:81631505-81632931 | 258 | 28.29 | 9.14 | 59.78 | 64.77 | -0.524 | Nucleus. |
| AslAA29 | AVESA.00010b.r2.5DG0993790.1 | Chr5D:81894570-81898210 | 255 | 27.65 | 5.85 | 60.12 | 66.98 | -0.485 | Nucleus. |
| AslAA30 | AVESA.00010b.r2.6AG1048590.1 | Chr6A:327663288-327665877 | 286 | 30.43 | 6.03 | 58.26 | 65.94 | -0.566 | Nucleus. |
| AslAA31 | AVESA.00010b.r2.6CG1127800.1 | Chr6C:98778482-98781263 | 293 | 31.02 | 6.05 | 56.4 | 67.68 | -0.533 | Nucleus. |
| AslAA32 | AVESA.00010b.r2.6DG1183280.2 | Chr6D:25787793-25791328 | 271 | 29.21 | 8.46 | 56.67 | 77.12 | -0.509 | Nucleus. |
| AslAA33 | AVESA.00010b.r2.6DG1151760.1 | Chr6D:283660830-283663860 | 285 | 30.28 | 6.16 | 58.41 | 65.47 | -0.582 | Nucleus. |
| AslAA34 | AVESA.00010b.r2.7AG1245870.1 | Chr7A:469862972-469866610 | 246 | 26.19 | 8.53 | 38.88 | 67.15 | -0.521 | Nucleus. |
| AslAA35 | AVESA.00010b.r2.7CG0702120.1 | Chr7C:47679337-47682257 | 233 | 24.86 | 8.92 | 45.72 | 64.59 | -0.543 | Nucleus. |
| AslAA36 | AVESA.00010b.r2.7CG0659560.1 | Chr7C:481809943-481813783 | 254 | 27.66 | 5.74 | 55.08 | 68.82 | -0.452 | Nucleus. |
| AslAA37 | AVESA.00010b.r2.7CG0659550.1 | Chr7C:482024536-482026340 | 218 | 23.37 | 8.28 | 60.42 | 60.6 | -0.491 | Nucleus. |
| AslAA38 | AVESA.00010b.r2.7CG0659540.1 | Chr7C:482194429-482195949 | 218 | 23.34 | 7.67 | 50.63 | 62.39 | -0.449 | Nucleus. |
| AslAA39 | AVESA.00010b.r2.7CG0659530.1 | Chr7C:482315736-482316778 | 265 | 29.16 | 9.23 | 61.94 | 65.62 | -0.511 | Nucleus. |
| AslAA40 | AVESA.00010b.r2.7DG1362020.1 | Chr7D:372085716-372089362 | 246 | 26.25 | 8.75 | 40.3 | 65.98 | -0.548 | Nucleus. |
