# Supplementary material for: Genome-wide unraveling of the AUX/IAA family in Avena sativa L. with observations on seedling root growth and stress resilience
Source: Front Plant Sci. 2026 Jun 9;17:1836152. doi: 10.3389/fpls.2026.1836152 (PMC13286757; doi:10.3389/fpls.2026.1836152)
Supplement: Supplementary Table 2 — Physicochemical properties of AsIAA genes. [file Table2.docx]

TableS3. Orthologs of AsIAA family genes in different species.

| Species gene ID | *Arabidopsis thaliana* | *Oryza sativa* | *Triticum aestivum* |
| --- | --- | --- | --- |
|  | AT5G20730 | Os06t0702600-01 | TraesCS6B02G167100.1 |
|  | AT1G19220 | Os02t0164900-02 | TraesCS2D02G491200.2 |
|  | AT2G01200 | Os06t0677800-01 | TraesCS2D02G548900.1 |
|  | AT1G15050 | Os06t0196700-02 | TraesCS6B02G141300.2 |
|  | AT1G19850 | Os12t0479400-02 | TraesCS7D02G161900.1 |
|  | AT5G57420 | Os04t0671900-01 | TraesCS2A02G309300.1 |
|  | AT4G32280 | Os02t0557200-01 | TraesCS7B02G316600.1 |
|  | AT4G14550 | Os04t0664400-02 | TraesCS7B02G363100.1 |
|  | AT3G23050 | Os02t0141100-00 | TraesCS7B02G138900.6 |
|  | AT3G04730 | Os12t0613700-01 | TraesCS3A02G159200.1 |
|  | AT1G04250 | Os04t0653300-00 | TraesCS2B02G519200.2 |
|  | AT5G65670 | Os01t0190300-01 | TraesCS2A02G547800.1 |
|  | AT2G22670 | Os05t0523300-01 | TraesCS6D02G102300.1 |
|  | AT4G29080 | Os01t0741900-01 | TraesCS5B02G039800.1 |
|  | AT5G43700 | Os02t0228900-01 | TraesCS5D02G045700.2 |
|  | AT4G14560 | Os11t0221000-01 | TraesCS2A02G491000.1 |
|  | AT3G23030 | Os11t0221300-00 | TraesCS7B02G065800.2 |
|  | AT1G04240 | Os11t0221200-00 | TraesCS7A02G461700.1 |
|  | AT1G80390 | Os08t0109400-01 | TraesCS6A02G138600.4 |
|  | AT1G15580 | Os06t0335500-01 | TraesCS3B02G475800.1 |
|  | AT1G52830 | Os05t0559400-01 | TraesCS3D02G166700.1 |
|  | AT3G15540 | Os01t0231000-03 | TraesCS7D02G449900.2 |
|  | AT3G62100 | Os05t0230700-01 | TraesCS6D02G100900.1 |
|  | AT2G46990 | Os01t0675700-01 | TraesCS7A02G416400.1 |
|  | AT3G17600 | Os03t0633800-01 | TraesCS6D02G100800.1 |
|  | AT2G33310 | Os07t0182400-01 | TraesCS7A02G252000.2 |
|  | AT1G04550 | Os12t0601400-01 | TraesCS7D02G409700.1 |
|  | AT1G04100 | Os03t0797800-01 | TraesCS2B02G578500.1 |
|  | AT4G28640 | Os05t0178600-01 | TraesCS6D02G127600.2 |
|  | AT3G16500 | Os01t0178500-02 | TraesCS6A02G113000.1 |
|  | AT1G51950 | Os02t0805100-01 | TraesCS5A02G038300.2 |
|  | AT5G25890 | Os12t0601300-01 | TraesCS7D02G250100.4 |
|  |  | Os01t0286900-01 | TraesCS2D02G506900.1 |
|  |  | Os03t0633500-01 | TraesCS2B02G534500.1 |
|  |  | Os02t0723400-02 | TraesCS2A02G506100.1 |
|  |  | Os03t0742900-01 | TraesCS3D02G276600.1 |
|  |  | Os06t0166500-01 | TraesCS1A02G122600.2 |
|  |  | Os02t0817600-02 | TraesCS3A02G276500.1 |
|  |  |  | TraesCS1D02G123500.1 |
|  |  |  | TraesCS3D02G110200.1 |
|  |  |  | TraesCS3A02G108400.1 |
|  |  |  | TraesCS3B02G310500.1 |
|  |  |  | TraesCS3B02G127400.1 |
|  |  |  | TraesCS1D02G345400.1 |
|  |  |  | TraesCS1B02G356600.1 |
|  |  |  | TraesCS1A02G343300.1 |
|  |  |  | TraesCS4A02G204900.1 |
|  |  |  | TraesCS4D02G105700.1 |
|  |  |  | TraesCS4B02G108700.1 |
|  |  |  | TraesCS7D02G318900.2 |
|  |  |  | TraesCS4B02G108600.2 |
|  |  |  | TraesCS4A02G205000.1 |
|  |  |  | TraesCS7B02G222900.1 |
|  |  |  | TraesCS7A02G322000.1 |
|  |  |  | TraesCS4D02G105800.1 |
|  |  |  | TraesCS7B02G242800.1 |
|  |  |  | TraesCS3D02G162700.1 |
|  |  |  | TraesCS7D02G339300.1 |
|  |  |  | TraesCS7A02G331100.1 |
|  |  |  | TraesCS3D02G269700.1 |
|  |  |  | TraesCS1B02G418400.1 |
|  |  |  | TraesCS5A02G058600.1 |
|  |  |  | TraesCS5A02G382600.1 |
|  |  |  | TraesCS1D02G084100.1 |
|  |  |  | TraesCS5B02G386800.1 |
|  |  |  | TraesCS5D02G388300.2 |
|  |  |  | TraesCS6B02G411000.1 |
|  |  |  | TraesCS5A02G317200.1 |
|  |  |  | TraesCS5D02G392000.1 |
|  |  |  | TraesCS5D02G323500.1 |
|  |  |  | TraesCS1A02G082700.2 |
|  |  |  | TraesCS6B02G432800.1 |
|  |  |  | TraesCS5B02G381900.1 |
|  |  |  | TraesCS5D02G069300.1 |
|  |  |  | TraesCS1B02G100300.1 |
|  |  |  | TraesCS6A02G392600.3 |
|  |  |  | TraesCS6A02G373300.1 |
|  |  |  | TraesCS5A02G378300.2 |
|  |  |  | TraesCS1A02G082700.1 |
