# Supplementary material for: Genome-wide unraveling of the AUX/IAA family in Avena sativa L. with observations on seedling root growth and stress resilience
Source: Front Plant Sci. 2026 Jun 9;17:1836152. doi: 10.3389/fpls.2026.1836152 (PMC13286757; doi:10.3389/fpls.2026.1836152)
Supplement: Supplementary Table 4 — Ct values and 2-ΔΔCT values of genes under different treatment conditions and in different tissues. [file DataSheet1.pdf]

Table S1. Primer information for qPCR used in this study.

Name	Forward Primer (5′->3′)	Reverse Primer (5′->3′)
AsIAA6	GCTCCAGGACAAGTTCATCTCA	TCTCCACAAACATTCTCCAGGG
AsIAA11	TGGAGTATGGAACGAGTTGCC	TTTCGGGAACAAAAGCGAACC
AsIAA13	GCTTCCTGTGTATGCCACTAGT	AGTATTGGCAGTACCCACCATG
AsIAA15	TTCGTCTCATGAAGAACTCCGAG	TAGATGAACCGATAAGGCGACAG
AsIAA17	AAAGGAGTAAATTGGCCTGTTGC	AGTTCAAGATTCGACACCACTGA
AsIAA19	CGCGAAAGTACTGGAAGCTTG	TAACCTCGCAACACGCAAAAG
AsEIF4A	TCTCGCAGGATACGGATGTCG	TCCATCGCATTGGTCGCTCT
